# Prognostic Value of Umbilical and Cerebral Doppler in Fetal Growth Restriction: Comparison of Dichorionic Twins and Singletons

**DOI:** 10.1371/journal.pone.0123067

**Published:** 2015-04-13

**Authors:** Sarah Vanlieferinghen, Olivia Anselem, Camille Le Ray, Yao Shen, Louis Marcellin, François Goffinet

**Affiliations:** 1 Department of Obstetrics and Gynaecology, Cochin Broca Hôtel-Dieu hospital, Assistance Publique-Hôpitaux de Paris, 53 avenue de l’observatoire, 75014 Paris, France; 2 PremUP Foundation for scientific cooperation in connection with pregnancy and prematurity, 53 avenue de l’observatoire, 75014 Paris, France; 3 French Institute of Health and Medical Research (INSERM) Unit 1153, Obstetrical, Perinatal and Pediatric Epidemiology Research Team (EPOPé), Research Center for Epidemiology and Biostatistics Sorbonne Paris Cité (CRESS), Paris, France; 4 Universitary Hospital Departement (DHU) Risks in pregnancy, Paris, France; University of Barcelona, SPAIN

## Abstract

**Objective:**

To compare the prognostic value of fetal Doppler in dichorionic twins and singletons by measuring the interval between diagnosis of an abnormal Doppler flow and birth in fetuses who are small for gestational age (SGA).

**Design:**

Comparative retrospective study using a prospectively collected database.

**Setting:**

A level 3 maternity unit in France.

**Population:**

Fetuses from singleton and dichorionic pregnancies who are SGA (vascular or unexplained), defined by an abdominal circumference (AC) measurement below the 10^th^ percentile and confirmed by a birth weight below the 10^th^ percentile.

**Methods:**

Fisher's exact and Chi-2 tests were used to compare frequencies, and the Mann-Whitney-Wilcoxon test was used to compare medians in non-Gaussian distributions.

**Main outcome measures:**

Both neonatal outcomes and intervals between the first Doppler abnormality and birth were compared in the groups of dichorionic twins and singletons.

**Results:**

Obstetric and neonatal outcome were similar in the 104 SGA dichorionic twins and 170 SGA singletons. Abnormalities of umbilical artery Doppler, regardless of type, appeared at the same frequency in both groups (52.9%) but were identified earlier in twins (25 versus 28 weeks, p = 0.02). Among fetuses with abnormal Doppler flow, the interval between the finding and birth was significantly longer in the twins than the singletons (44 vs 15 days, p<0.01).

**Conclusions:**

The prognostic value of an abnormal Doppler finding for the course of a pregnancy may be different in dichorionic twins and singletons. The management of women carrying SGA twins and the information provided to them should take these results into account.

## INTRODUCTION

Many studies have examined the pathophysiology and outcome of singletons born small for gestational age (SGA) due to vascular causes. Monitoring procedures in these cases are relatively consensual, as are the factors used to decide upon early intervention for delivery: fetal heart rate (FHR) abnormalities, arrested growth, estimated fetal weight, and Doppler flow abnormalities. In particular, fetal Doppler has proved its value in monitoring these fetuses [1] and plays an important role in decisions about early delivery.

Twins are more frequently SGA than singletons [2]. Data are sparse about the prognostic value of fetal Doppler when one or both twins are SGA. In view of its elevated prognostic value in singletons, fetal Doppler is recommended in monitoring and deciding on delivery in these cases [3]. The pathophysiology of abnormal growth in twins, however, may differ from that in singletons and may be related to abnormalities of blood flow distribution and of placentation [4]. It is therefore possible that the prognostic value of fetal Doppler examinations differs from that observed in singletons. If so, their use in monitoring and making medical decisions should be adapted to this particular population of twins.

We compared the prognostic value of fetal Doppler in SGA dichorionic twins and singletons by measuring the time interval between an abnormal Doppler finding and birth.

## METHODS

### Study population

This retrospective study was conducted in a level 3 maternity unit in France. The group of twins included all fetuses in twin pregnancies prenatally diagnosed as SGA born between January 1, 2007, and December 31, 2010. When both twins from the same pregnancy were diagnosed as SGA, both were included. The group of singletons included all fetuses in singleton pregnancies prenatally diagnosed as SGA born between January 1, 2008, and December 31, 2009. A longer period was chosen for recruitment of twins in view of the lower incidence of twin pregnancies.

The guidelines in our department are based on Clinical Practice Guidelines issued by the French College of Gynecologists and Obstetricians (CNGOF, [5], see www.cngof.asso.fr).

The cases were identified with DIAMM software (MICRO6 SARL, version 7.3.7 Rev2, Villiers Les Nancy, France) from a prospectively collected and recorded database of information about all pregnancies in our hospital. To verify the inclusion and exclusion criteria, we examined the case records of all fetuses who had a measurement at or below the 10^th^ percentile according to the curves of the French College of Fetal Echography used by DIAMM software, for any relevant biometric indicator (biparietal diameter, head circumference, abdominal circumference (AC), or femur length) [6] and all children with a birth weight at or below the 10^th^ percentile according to the curves of Leroy and Lefort [7].

SGA status was defined by at least one ultrasound AC measurement at or below the 10^th^ percentile according to the fetal biometry curves used in our maternity ward [8]. It was defined as severe if the AC was below the 3^rd^ percentile and moderate if between the 3^rd^ and 10^th^ percentiles [9].

We excluded fetuses with a birth weight above the 10^th^ percentile. Cases were also excluded from the analysis when SGA status was associated with a malformation, a karyotype anomaly, fetomaternal infection, fetomaternal alloimmunization, or premature rupture of the membranes before 24 weeks. Because of their specific complications, monochorionic twin pregnancies were excluded, as were multiple pregnancies that resulted in a twin pregnancy after embryo reduction.

### Management of SGA fetuses

Monitoring was similar for twins and singletons suspected to be SGA. Ultrasound monitoring included weekly measurements of the umbilical artery Doppler (UAD) and cerebral artery Doppler indices and measurement of the biometric indicators mentioned above every 2 weeks by the same operator. UAD measurements were taken at the level of the perivesical umbilical artery or its abdominal insertion for twins and at the level of the free loop of the umbilical artery for singletons. Depending on the severity of the SGA status and the Doppler abnormalities, FHR was monitored at intervals ranging from weekly to twice daily, with analysis of the short-term variability.

The decision about time and mode of delivery was based on the same set of factors in twins and singletons. These included analysis of the FHR, fetal growth, Doppler abnormalities, gestational age, estimated fetal weight, and signs of maternal decompensation.

Generally, the basis for decisions about time of delivery was principally arrested growth or FHR abnormalities [10]. Delivery was never based only on Doppler abnormalities. Arrested growth was defined as the failure of either AC or estimated fetal weight to increase between two examinations performed at least 10 days apart. The FHR abnormalities that led to immediate intervention for delivery were repeated or prolonged decelerations, or a micro-oscillating rhythm, or a reduction in short-term variability (< 3 ms). Intervention was not considered below 24 weeks of gestation or an estimated fetal weight below 500 grams in dichorionic twins or singletons.

### Factors studied

The general and obstetric characteristics were: maternal age, parity, geographic origin, pre-pregnancy body mass index (BMI), fetal sex, gestational age, and AC measurement (in millimeters and percentiles), as well as abnormalities of the UAD and cerebral artery Doppler observed at SGA diagnosis. The UAD was classified as normal or as abnormal with either positive diastolic flow or absent or reverse diastolic flow. It was defined as abnormal with positive diastolic flow when the resistance index of the umbilical artery (S-D/S) ranged from 0.80 to 0.99, regardless of gestational age. We defined a group with any UAD abnormalities, including the cases that were abnormal with positive diastolic flow and those with absent or reverse diastolic flow. When the abnormalities continued to increase in severity, we analyzed the earliest one. An abnormal cerebroplacental ratio was defined by a resistance index higher for the UAD than for the cerebral artery Doppler. Time between the first Doppler abnormality and birth was the principal outcome measure.

The obstetric outcome data collected were mode of delivery and indications for early intervention for delivery, defined as cesarean before labor or induction of labor.

A composite criterion defining poor neonatal condition was fulfilled by any one of the following criteria: 5-min Apgar score ≤ 7, umbilical cord arterial pH ≤ 7.20, NICU admission, or neonatal death. Other neonatal outcomes were gestational age at delivery, or birth weight (in grams and percentiles according to the Leroy and Lefort curves [7]). *In utero* fetal death (IUFD) was defined by spontaneous fetal death after the first trimester of pregnancy, i.e., after 14 weeks of gestation. Medically indicated termination of pregnancy occurred in some extremely SGA fetuses. The decision to terminate the pregnancy was based primarily on the parents’ request but also on a set of factors including gestational age less than 24 weeks or estimated fetal weight below 600 grams, arrested growth, and reverse diastolic flow on the UAD especially with an associated risk of serious maternal complications (severe preeclampsia).

Preeclampsia was defined by a systolic blood pressure above 140 mm Hg and a diastolic pressure greater than 90 mm Hg, taken at rest, at least twice, associated with proteinuria greater than 0.3 g/24 h after 20 weeks of gestation [11].

### Statistical analysis

We compared the twins and singletons for frequency of abnormal Doppler findings, gestational age at this observation, neonatal outcome, and interval between the first Doppler abnormality and birth. The comparison of the interval to birth took into account how severely the fetus was SGA at diagnosis.

Fisher's exact and Chi-2 tests were used to compare frequencies (GraphPad Prism software, version 5.00, GraphPad Software, San Diego CA, www.graphpad.com), and the Mann-Whitney-Wilcoxon test was used to compare medians in non-Gaussian distributions (Macro of nonparametric tests, AstroResearch©, July, 2003). Differences were considered significant when p< 0.05.

### Ethics statement

Patient data were anonymized and de-identified before analysis.

This study was approved by the National Data Protection Authority (Commission Nationale de l’Informatique et des Libertés, CNIL n° 1755849).

## RESULTS

The rate of dichorionic twin pregnancies was fairly stable over the study period: 4.0% in 2007, 4.5% in 2008, 3.3% in 2009, and 4.9% in 2010. The electronic database contained 234 potential SGA cases in twins and 643 in singletons. Examination of each case record showed that 89 records of dichorionic twin pregnancies, including 15 in which both twins were SGA, for a total of 104 fetuses, and 170 records of singletons (control group) met the inclusion and exclusion criteria.

The mothers of dichorionic twins were older (35 vs 32.5 years, p<0.01) and had a lower BMI (BMI = 20.7 vs 22, p = 0.01). An AC below the 3^rd^ percentile (severe SGA status) at diagnosis was less frequent in twins than in singletons (9.6% vs 20.6%, p = 0.02) (Table 1). Eight twins and eight singletons died *in utero* (7.7 vs 4.7%, p = 0.43). Moreover, pregnancy was terminated for 13 very severely SGA fetuses, all in the singleton group. The rate of preeclampsia was 23.0% (n = 24) in women with twins and 46.5% (n = 79) in women with singletons, p<0.01).

**Table 1:** Maternal and fetal characteristics at diagnosis at diagnosis at SGA.

		Twins N = 104	Singletons N = 170	p
Maternal age in years, median [25th;75th percentile]		35 [31.5; 39.0]	32.5 [28.0; 37.5]	<0.01
Nulliparas n (%)		72 (69.2)	104 (61.2)	0.20
Place of birth n (%):				
	Europe	70 (67.3)	93 (54.7)	
	Africa	27 (26)	62 (36.5)	
	North & South America	3 (2.9)	5 (2.9)	
	Asia	4 (3.8)	10 (5.8)	0.22
BMI (before pregnancy) kg/m2 median [25th; 75th percentile]		20.7 [18.9; 23.2]	22 [19.5; 25.4]^a^	0.01
Male n (%)		43 (41.3)	75 (44.1)	0.70
Gestational age in weeks median [25th; 75th percentile]		29 [24; 34]	28 [25; 32]	0.92
AC in mm, median [25th; 75th percentile]		227 [175.0; 260.0]	209 [178.2; 242.5]	0.22
AC < 3^rd^ p n (%)		10 (9.6)	35 (20.6)	0.02
UAD abnormalities n (%)		35 (33.6)	56 (32.9)	0.90
Abnormal cerebroplacental ratio n (%)		22 (21.2)	35 (20.6)	1

^a^ N = 162

BMI: Body mass index, AC: Abdominal circumference, UAD: umbilical artery Doppler, weeks: weeks of gestation

Composite “UAD abnormalities “criterion: abnormal umbilical Doppler, absent or reverse flow

Table 2 describes mode of delivery and neonatal condition for live births (96 twins and 149 singletons). Cesarean delivery was less frequent in the dichorionic twins (64.6% vs 77.9%, P = 0.03). Gestational age at delivery and median birth weight were lower in singletons than in twins (33.6 vs 35.4 weeks, p = 0.02 and 1380 vs 1835 grams, p = 0.01). Moreover indications for cesarean before labor or induction were different between the groups, and there were more FHR abnormalities among the singletons than for twins (32.2% vs 11.5%, p<0.01). The rate of early intervention for delivery for preeclampsia was the same in both groups (18.7% (n = 18) vs 18.8% (n = 28), p = 1.00).

**Table 2:** Mode of delivery and neonatal condition for live births.

		Twins N = 96	Singletons N = 149	p
Interval between diagnosis and birth in days, median [25th; 75th percentile]				
	Total	24.5 [11.0; 51.7]	21.0 [5.0; 45.0]	0.12
	Severely SGA (< 3^rd^ p)	19.0 [15.0; 30.7]	14.5 [4.7; 48.2]	0.16
	Moderately SGA (3–10^th^ p)	25.0 [10.2; 54]	24.0 [88.0; 46.5]	0.30
Mode of labor onset n (%)				
	Spontaneous labor	15 (15.6)	29 (19.5)	
	Induction	28 (29.2)	7 (4.7)	
	Cesarean before labor	55 (55.2)	113 (75.8)	<0.01
Indications for early intervention for delivery^a^ n (%)				
	Arrested growth	20 (20.8)	32 (21.5)	1.00
	FHR abnormality	11 (11.5)	48 (32.2)	<0.01
	Maternal	20 (20.8)	30 (20.1)	1.00
	UAD abnormalities	9 (9.4)	4(2.7)	0.04
	Other^b^	29 (30.2)	10 (6.7)	<0.01
Cesareans (total) n (%)		62 (64.6)	116 (77.9)	0.03
Gestational age at delivery in weeks, median [25th; 75th percentile]				
	Total	35.4 [33.5; 37.3]	33.6 [29.5; 37.5]	0.02
	< 33 weeks n (%)	19 (19.8)	66 (44.3)	
	33 weeks n (%)	42 (43.7)	38 (25.5)	
	≥ 37 weeks n (%)	35 (36.4)	45 (30.2)	<0.01
Birth weight in grams median [25th; 75th percentile]		1835 [1327; 2065]	1380 [875; 2100]	0.01
Birth weight < 3^rd^ p n (%)		54 (56.2)	74 (49.7)	0.36
Birth weight ≤ 10^th^ p n (%)		96 (100)	146 (98.0)	0.28
5-min Apgar < 7 n (%)^c^		6 (6.3)	14 (9.7)	0.48
pH ≤ 7.20 n (%)^d^		16 (17.2)	24 (16.4)	0.86
Admission to NICU n (%)		79 (82.3)	117 (78.5)	0.52
In-hospital neonatal mortality n (%)		2 (2)	2 (1.3)	0.65
Poor neonatal condition n (%)		81 (84.4)	126 (84.6)	1.00

weeks: weeks of gestation; composite "poor neonatal condition" criterion: 5-min Apgar ≤ 7 or pH < 7.20 or admitted to NICU or died in the delivery room

^a^ early intervention for delivery: multiple indications possible for induction or cesareans

^b^ premature rupture of the membranes, post-term, threatened preterm delivery, contraindication to vaginal delivery, indication for second twin

^c^ missing data for 5: 1 twin, 4 singletons

^d^ missing data for 3 in each group

The rate of poor neonatal condition did not differ between the groups (84.4% in twins vs 84.6% in singletons, p = 1.0), nor did the other neonatal outcomes (Table 2).

The frequency of UAD abnormalities with positive diastolic flow was similar in both groups but was diagnosed earlier in twins than singletons (mean of 25 vs 28 weeks, p = 0.02). The interval between the first abnormal UAD finding with a positive diastolic flow and birth was substantially longer in twins than singletons (53 vs 16 days, p<0.01). This longer interval in twins was found both in severely and moderately SGA fetuses (Table 3).

**Table 3:** Comparison of fetal Doppler flow abnormalities, gestational age at their onset, and interval between onset and birth.

			Twins N = 104	Singletons N = 170	p
**Abnormal UAD with positive diastolic flow**	n (%)		35 (33.7)	63 (37.0)	0.60
	Gestational age at onset, in weeks median [25th; 75th percentile]		25.0 [23.5; 29.5]	28.0 [25.0; 32.0]	0.02
	Interval until birth, d, median [25th;75th percentile]	total	53.0 [34.0; 84.0]	16.0 [6.0; 34.0]	<0.01
		severe SGA (< 3 ^rd^ p)	85.0 [69.0; 88.0]	20.0 [5.0; 33.5]	0.01
		moderate SGA (3–10^th^ p)	49.0 [31.7; 76.5]	16.0 [6.0; 34.0]	<0.01
**UAD Absent diastolic or reverse flow**	n (%)		45 (43.3)	54 (31.8)	0.07
	Gestational age at onset, in weeks, median [25th; 75th percentile]		28.0 [25.0; 32.0]	26.5 [24.0; 29.0]	0.06
	Interval until birth, d, median [25th; 75th percentile]	total	23.0 [6.0; 45]	12.0 [4.0; 26.7]	0.05
		severe SGA (< 3^rd^ p)	23.0 [13.5; 39.0]	9.0 [8.0; 24.0]	0.09
		moderate SGA (3–10^th^ p)	20.5 [5.2; 44.7]	12.0 [2.2; 28.5]	0.12
**UAD abnormalities**	n (%)		55 (52.9)	90 (52.9)	1.00
	Gestational age at onset, in weeks, median [25th; 75th percentile]		25.0 [23.5; 29.0]	28.0 [25.0; 30.0]	0.02
	Interval until birth, d, median [25th; 75th percentile]	total	44.0 [26.0; 73.0]	15.0 [5.0; 31.0]	<0.01
		severe SGA (< 3^rd^ p)	56.0 [32.7; 78.5]	15.0 [4.0; 31.0]	0.03
		moderate SGA (3–10^th^ p)	43.0 [27.0; 72.0]	15.0 [5.0; 33.0]	<0.01
**Abnormal cerebroplacental ratio**	n (%)		51 (49.0)	75 (44.1)	0.46
	Gestational ageat onset, in weeks, median [25th; 75th percentile]		30.0 [27.0; 33.5]	30.0 [27.0; 34.0]	0.68
	Interval until birth d, median [25th; 75th percentile]	total	26.0 [10.5; 36.5]	8.0 [2.0; 18.5]	<0.01
		severe SGA (< 3^rd^ p)	26.0 [21.5; 29.5]	9.0 [8.5; 9.5]	0.09
		moderate SGA (3–10^th^ p)	24.0 [9.0; 37.0]	7.5 [3.0; 16.2]	<0.01

The analysis covers all subjects, including the *in utero* deaths

AC: abdominal circumference, UAD: umbilical artery Doppler, F: female, M: male, d: days

Composite "UAD abnormalities" criterion: any UAD abnormality, including abnormal UAD with positive diastolic flow and absent or reverse diastolic. Calculation of interval until birth: number of days from the abnormal Doppler findings to the birth.

UAD with absent diastolic or reverse flow was found at the same frequency and diagnosed at the same gestational age in both groups. The interval between the UAD finding of absent diastolic or reverse flow and birth was 23 days in twins and 12 days in singletons (p = 0.05); when found between 26 and 34 weeks, the pregnancy continued after 34 weeks for 9 twins and 3 singletons.

The frequency of all types of abnormal UAD findings, like that of positive diastolic flow, was similar in both groups and occurred earlier in twins than in singletons (25 vs 28 weeks, p = 0.02). The interval between the first abnormal UAD finding and birth was notably longer in twins than in singletons (44 vs 15 days, p<0.01), even after stratification of fetuses as moderately or severely SGA at diagnosis.

The frequency of an abnormal cerebroplacental ratio was similar in both groups and was diagnosed at the same gestational age in twins and singletons (mean of 30.0 weeks, p = 0.68). The interval between this finding and birth was longer in twins than singletons (26 vs 8 days, p<0.01).

## DISCUSSION

### Main Findings

The abnormalities of umbilical artery and cerebral artery Doppler results in SGA fetuses do not appear to have the same prognostic significance for the course of the pregnancy in dichorionic twins and singletons. In particular, the interval between an abnormal Doppler finding and birth is significantly longer in twins than in singletons.

### Strengths and Limitations

Some maternal characteristics were not distributed comparably in the two study groups. As expected, mothers of twins were older and more often of European origin; both factors are explained by the high proportion of twin pregnancies involving assisted reproductive technology. Moreover, older maternal age is associated with a higher frequency of spontaneous multiple pregnancies [12]. Women carrying singletons had a higher BMI than those pregnant with twins. This finding may be explained by an over-representation of African women in the singleton group (the median BMI of the African women was higher than that of the European women (23 [20.8; 26.4] vs 20.6 [18.9; 23], p<0.0001) and by a tendency in France to not perform ART for obese women. It is improbable that the different distribution of these factors in the two groups explains the difference observed in the interval between the abnormal Doppler findings and birth.

Because our protocols for management in terms of monitoring and medical decision-making once a fetus is suspected to be SGA are the same for singletons and twins, indication bias should be limited. It is nonetheless possible that the health of the co-twin influenced some medical decisions toward an earlier or later intervention for delivery, but we were unable to take this information into account in this analysis. Moreover, in dichorionic twin pregnancies with one severely SGA fetus, no selective terminations of pregnancy were performed, to avoid complications of this invasive procedure for the healthy co-twin.

Furthermore, the fact that we used resistance indices for Doppler abnormalities, regardless gestational age, instead of pulsatility indices which varies with gestational age, could be a limitation for the external validity of the study. However there is no proof of the superiority of the IP compared to IR so French guidelines for SGA and FGR do not recommend one over another index [5].

### Interpretation

Singletons were severely SGA more frequently than twins. It is possible that the routine monthly ultrasound monitoring of twins enabled earlier SGA diagnoses and detection of Doppler abnormalities, before severe growth impairment could occur. To limit the effect of this bias on the intervals we analyzed, we performed the principal comparisons while stratifying the populations as severe and moderate SGA, which allowed us to confirm that the results for these intervals were the same in both strata. Moreover, both the percentage of newborns with a birth weight below the 3^rd^ percentile and the mean weight were similar in the two groups. Neonatal condition was also similar in both groups.

The proportion of Doppler abnormalities, regardless of type, at diagnosis or during the monitoring, was similar in both groups but there was a trend toward more UAD findings of absent diastolic or reverse flow in twins. It is recommended that UAD measurements be taken at the level of the perivesical umbilical artery or its abdominal insertion for twins and at the level of the free loop of the umbilical artery for singletons [13]. Accordingly, resistance measurements might have been slightly overestimated in twins compared with singletons. This could have led to overestimation of the intervals between UAD diagnosis and birth for twins.

The objective of the UAD measurement in fetuses suspected to be SGA is to adapt monitoring accordingly, to predict the onset of unfavorable outcome, and finally to improve health, as this measurement has been demonstrated to do in singletons [14]. This examination has thus become essential in monitoring and making decisions about fetuses suspected to be SGA, even for abnormalities that if isolated would not be used as decisional factors. The published studies on this specific question in twins are discordant, old, and include monochorionic pregnancies, all factors that make them difficult to interpret [14]. Of 18 trials about the utility of UAD measurements included in a Cochrane meta-analysis, only three included twin pregnancies. While the use of Doppler imaging in singletons suspected to be SGA has made it possible to reduce perinatal mortality (RR = 0.71, 95% CI [0.52–0.98]), it is not yet possible to draw conclusions about its value in twin pregnancies[15–17].

Accordingly, the results from UAD and cerebral artery Doppler examinations should perhaps be used differently in SGA dichorionic twins and SGA singletons for decisions concerning their monitoring and operative intervention. The longer interval we observed between the onset of Doppler abnormalities and birth in twins did not result in an increase in FHR abnormalities, emergency operative intervention, or poor neonatal condition.

## CONCLUSION

Abnormalities of umbilical artery Doppler and abnormal cerebroplacental ratios in cases of SGA do not appear to have the same prognostic significance for the course of the pregnancy in dichorionic twins and singletons. The intervals between the abnormal Doppler findings and birth are longer in the twins. These results suggest that methods of monitoring and decision-making following these findings may be different for these two groups. The information provided to women carrying twins about the expected time until birth after such findings should take these results into account, particularly if Doppler abnormalities are involved in the decision for early delivery.
